# Supplementary material for: Shared Treg dysregulation underlies psoriasis vulgaris and atopic dermatitis through the PHF19-PRC2 epigenetic axis
Source: Front Immunol. 2026 Aug 20;17:1932788. doi: 10.3389/fimmu.2026.1932788 (PMC13538097; doi:10.3389/fimmu.2026.1932788)
Supplement: Supplementary file 3 [file DataSheet3.zip › Additional files/Additional file 2/58.FAM200B.forest.pdf]

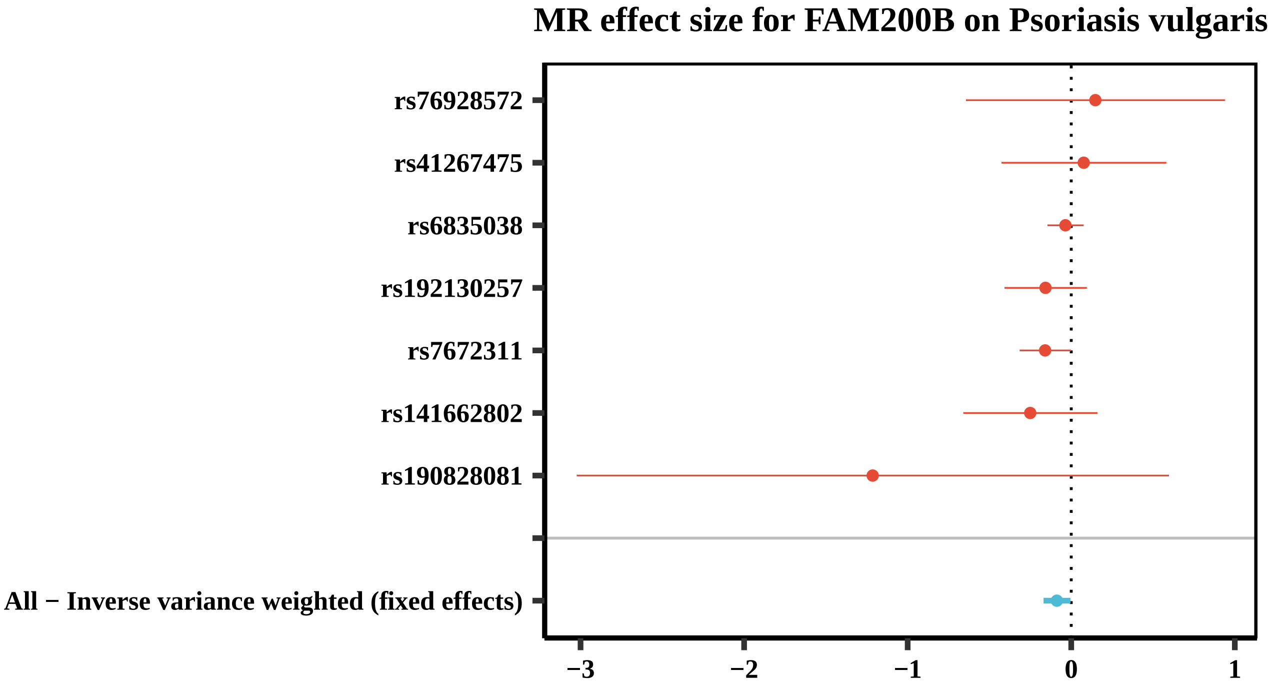

MR effect size for FAM200B on Psoriasis vulgaris
rs76928572
rs41267475
rs6835038
rs192130257
rs7672311
rs141662802
rs190828081
All − Inverse variance weighted (fixed effects)
−3
−2
−1
0
1
